# Supplementary material for: Chronic glucose-dependent insulinotropic polypeptide receptor (GIPR) agonism desensitizes adipocyte GIPR activity mimicking functional GIPR antagonism
Source: Nat Commun. 2020 Oct 5;11:4981. doi: 10.1038/s41467-020-18751-8 (PMC7536395; doi:10.1038/s41467-020-18751-8)
Supplement: Supplementary file 1 — Supplementary Information [file 41467_2020_18751_MOESM1_ESM.pdf]

<u>Supplemental Information</u> for "Chronic glucose-dependent insulinotropic polypeptide receptor (GIPR) agonism desensitizes adipocyte GIPR activity mimicking functional GIPR antagonism"

Supplemental Figures 1-5 and Legends…page 2
Supplemental Table 1 and Legend……….page 7
Supplemental Data 1 Legend………………..page 7

Supplemental Figure 1

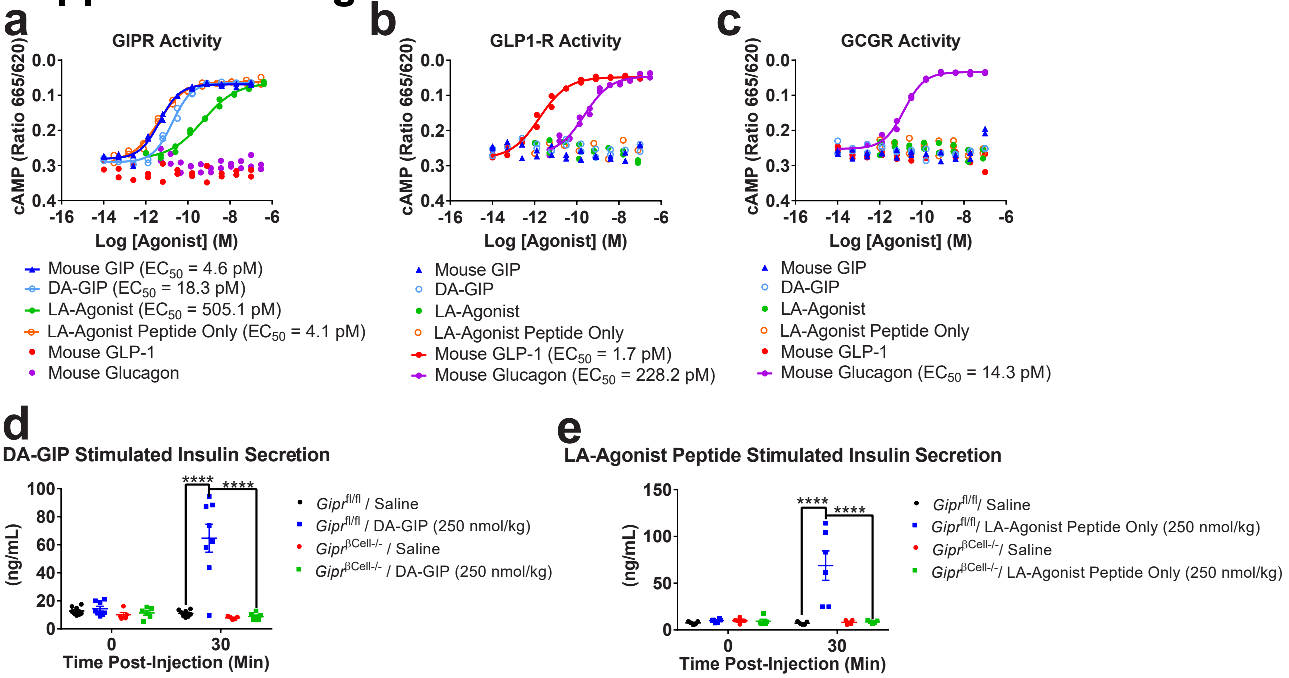

Supplemental Figure 1. The long-acting GIPR agonist is specific for GIPR.

(A-C) cAMP measurements of agonist activity of mouse GIP, DA-GIP, LA-Agonist, LA-Agonist peptide only, mouse GLP-1, and mouse glucagon in cell lines overexpressing (A) GIPR, (B) GLP1-R, or (C) glucagon receptor. n = 2 wells/treatment.

(D) *Gipr*$^{fl/fl}$ and *Gipr*$^{\beta Cell-/-}$ mice fed HFD for 18 weeks were fasted for 6 hours then RO bled for T0, immediately IP injected with glucose (0.5 g/kg) and saline or glucose (0.5 g/kg) and DA-GIP (250 nmol/kg), then blood collected by RO bleed after 30 min. Plasma insulin was measured by ELISA. n = 8 mice/group *Gipr*$^{fl/fl}$ / Saline and *Gipr*$^{fl/fl}$ / DA-GIP, n = 5 mice *Gipr*$^{\beta Cell-/-}$ / Saline, and n = 6 mice *Gipr*$^{\beta Cell-/-}$ DA-GIP; ****$p < 0.0001$, two-way repeated measure ANOVA with Tukey's HSD for multiple comparisons. Data are presented as mean values +/- SEM.

(E) *Gipr*$^{fl/fl}$ and *Gipr*$^{\beta Cell-/-}$ mice fed HFD for 8 weeks were fasted for 6 hours then RO bled for T0, immediately IP injected with glucose (0.5 g/kg) and saline or glucose (0.5 g/kg) and LA-Agonist peptide only (250 nmol/kg), then blood collected by RO bleed after 30 min. Plasma insulin was measured by ELISA. n = 5 mice *Gipr*$^{fl/fl}$ / Saline, n = 6 mice *Gipr*$^{fl/fl}$ / LA-Agonist Peptide Only, n = 4 mice *Gipr*$^{\beta Cell-/-}$ / Saline, and n = 5 mice *Gipr*$^{\beta Cell-/-}$ LA-Agonist Peptide Only; ****$p < 0.0001$, two-way repeated measure ANOVA with Tukey's HSD for multiple comparisons. Data are presented as mean values +/- SEM.

Supplemental Figure 2

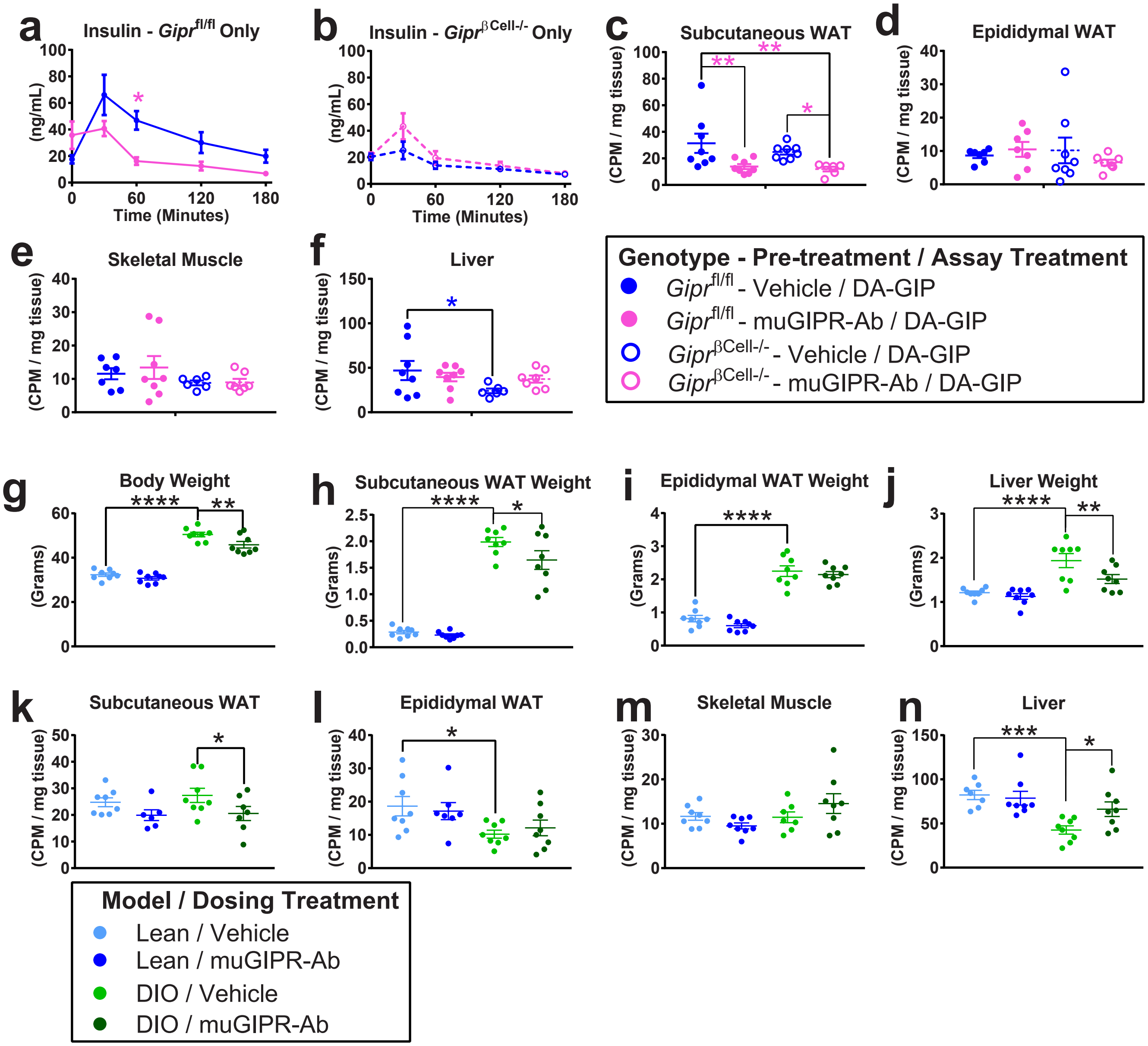

Supplemental Figure 2. GIP stimulated FA uptake into adipose tissue is not dependent on GIP-stimulated insulin secretion and is inhibited after chronic muGIPR-Ab treatment.

(A-F) *Gipr*$^{fl/fl}$ and *Gipr*$^{\beta Cell-/-}$ mice fed HFD for 13 weeks pre-treated with a single injection of vehicle or muGIPR-Ab (25 mg/kg) for 24 hours then injected with DA-GIP and simultaneously oral gavaged with 14C-oleic acid in olive oil to assess FA uptake. Plasma insulin measured over time in (A) *Gipr*$^{fl/fl}$ mice and (B) *Gipr*$^{\beta Cell-/-}$ mice, *p = 0.020. Radioactivity uptake into metabolically relevant tissues was measured at necropsy 180 min post-dose in (C) scWAT, (D) eWAT, (E) skeletal muscle, and (F) liver; (C) *Gipr*$^{fl/fl}$/DA-GIP vs. *Gipr*$^{fl/fl}$/muGIPR-Ab + DA-GIP **p = 0.0054, *Gipr*$^{fl/fl}$/DA-GIP vs. *Gipr*$^{\beta Cell-/-}$/muGIPR-Ab + DA-GIP **p = 0.0044, *Gipr*$^{\beta Cell-/-}$/DA-GIP vs. *Gipr*$^{\beta Cell-/-}$/muGIPR-Ab + DA-GIP *p = 0.49; (F) *Gipr*$^{fl/fl}$/DA-GIP vs. *Gipr*$^{\beta Cell-/-}$/DA-GIP *p = 0.032. (A) n = 8 mice/group, (B) n = 7 mice/group, (C-F) n = 8 *Gipr*$^{fl/fl}$ mice/group and n = 7 *Gipr*$^{\beta Cell-/-}$ mice/group; (A, B) two-way repeated measures ANOVA with Sidak's test for multiple comparisons or (C-F) one-way ANOVA with Fisher's posthoc test, *p < 0.05 and **p < 0.01. Data are presented as mean values +/- SEM.

(G-N) Lean and DIO mice treated with vehicle or muGIPR-Ab (25 mg/kg every 6 days) for 41 days then injected with DA-GIP and simultaneously oral gavaged with 14C-oleic acid in olive oil to assess FA uptake. (G) Body weight, (H) scWAT weight, (I) eWAT weight, and (J) liver weight at necropsy; (G) ****p < 0.0001 and **p = 0.0045, (H) ****p < 0.0001 and *p = 0.023, (I) ****p < 0.0001, and (J) ****p < 0.0001 and **p = 0.0066. Radioactivity uptake into metabolically relevant tissues was measured at necropsy 180 min post-dose in (K) scWAT, (L) eWAT, (M) SKM, and (N) liver. (K) *p = 0.045, (L) *p = 0.015, and (N) ***p = 0.0004 and *p = 0.019. One-way ANOVA with Fisher's posthoc test, *p < 0.05, **p < 0.01, ***p < 0.001, and ****p < 0.0001. Data represent means +/- SEM of n = 8 mice/group.

Supplemental Figure 3

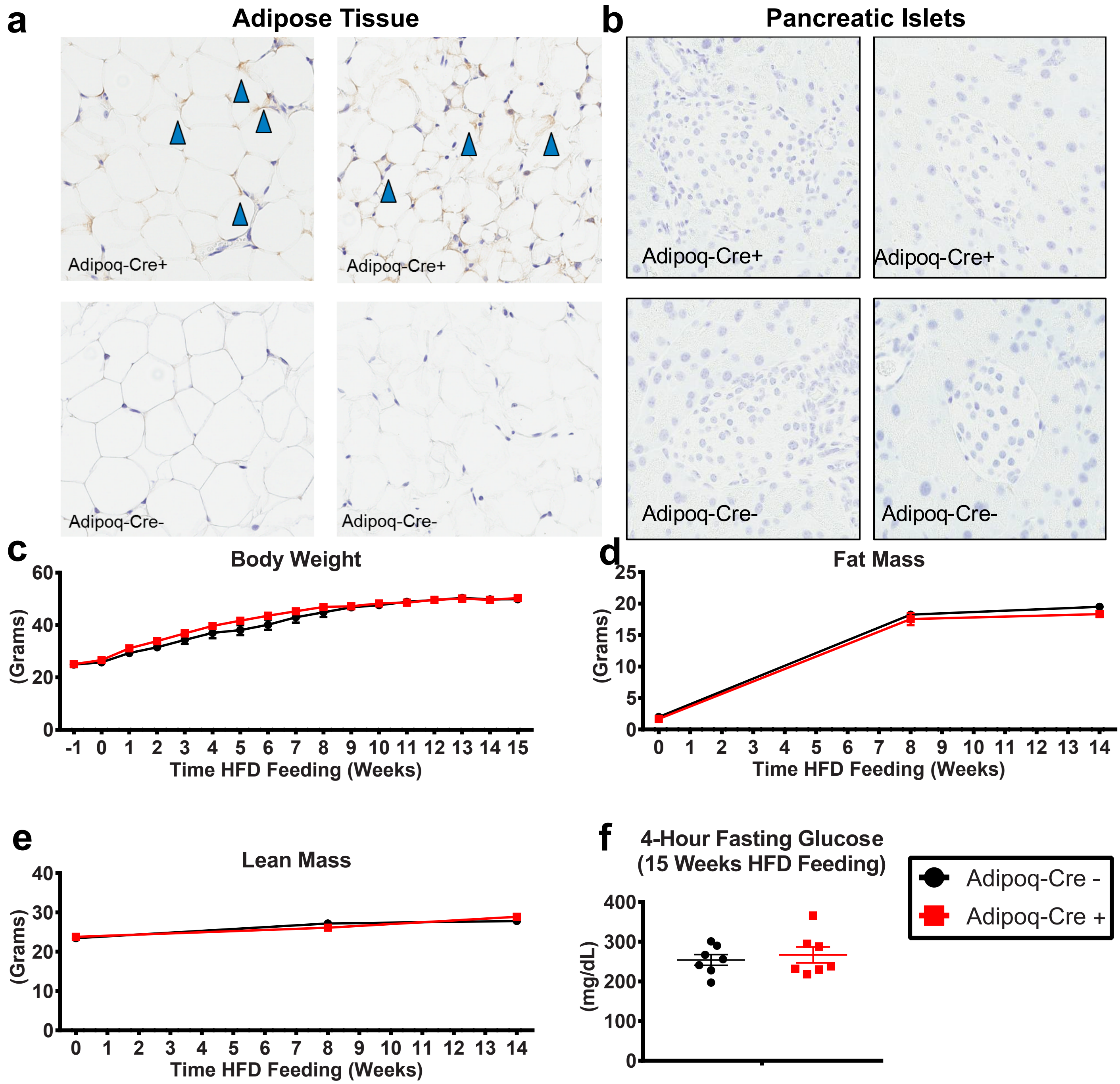

Supplemental Figure 3. Phenotype of adiponectin-Cre mice.

(A-B) Representative images of β-galactosidase staining in (A) adipose tissue and (B) pancreatic islets of mice produced from mating R26R x Adipoq-Cre mice to confirm Adipoq-Cre mice specifically express Cre recombinase adipose tissue and not in pancreatic islets (negative control). Reproductive tissues also were assessed, and no staining was detected (data not shown).

(C-F) Male Adipoq-Cre- and Adipoq-Cre+ littermates fed HFD for 15 weeks. (C) Body weight, (D) fat mass, and (E) lean mass measured over time. (F) 4-hour fasting blood glucose from terminal decapitation blood collection after 15 weeks HFD feeding. n = 7 mice/group. Data are presented as mean values +/- SEM.

Supplemental Figure 4

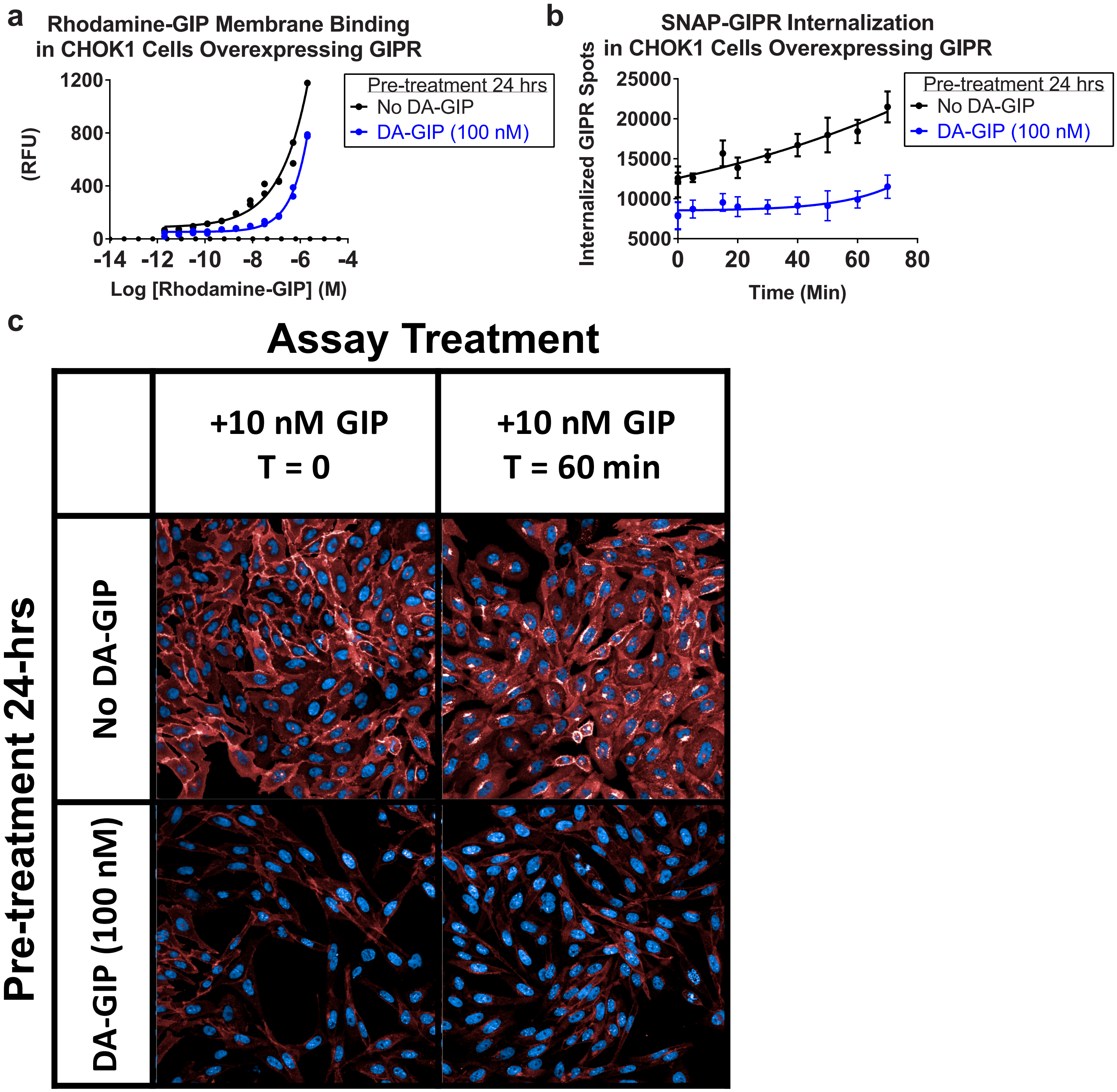

Supplemental Figure 4. Reduced plasma membrane GIPR expression after chronic GIP treatment.

(A) Rhodamine intensity measurement of CHOK1+GIPR cells pre-treated with or without DA-GIP (100 nM) for 24 hours followed by incubation with rhodamine-GIP on ice for 60 min; n = 2 wells/treatment.

(B-C) Time course comparison of GIPR internalization upon stimulation with GIP (10 nM) in CHOK1+SNAP-GIPR cells labeled with cell impermeable SNAP-Surface Alexa Fluor 647 substrate that only adds the fluorescent tags to GIPR on the plasma membrane. Prior to labeling, cells were pre-treated with or without DA-GIP (100 nM) for 24 hours. Quantitated membrane GIPR fluorescence (RFU) with background fluorescence subtracted (B) and representative images (C), n = 4 wells/treatment. Data are presented as mean values +/- SEM.

Supplemental Figure 5

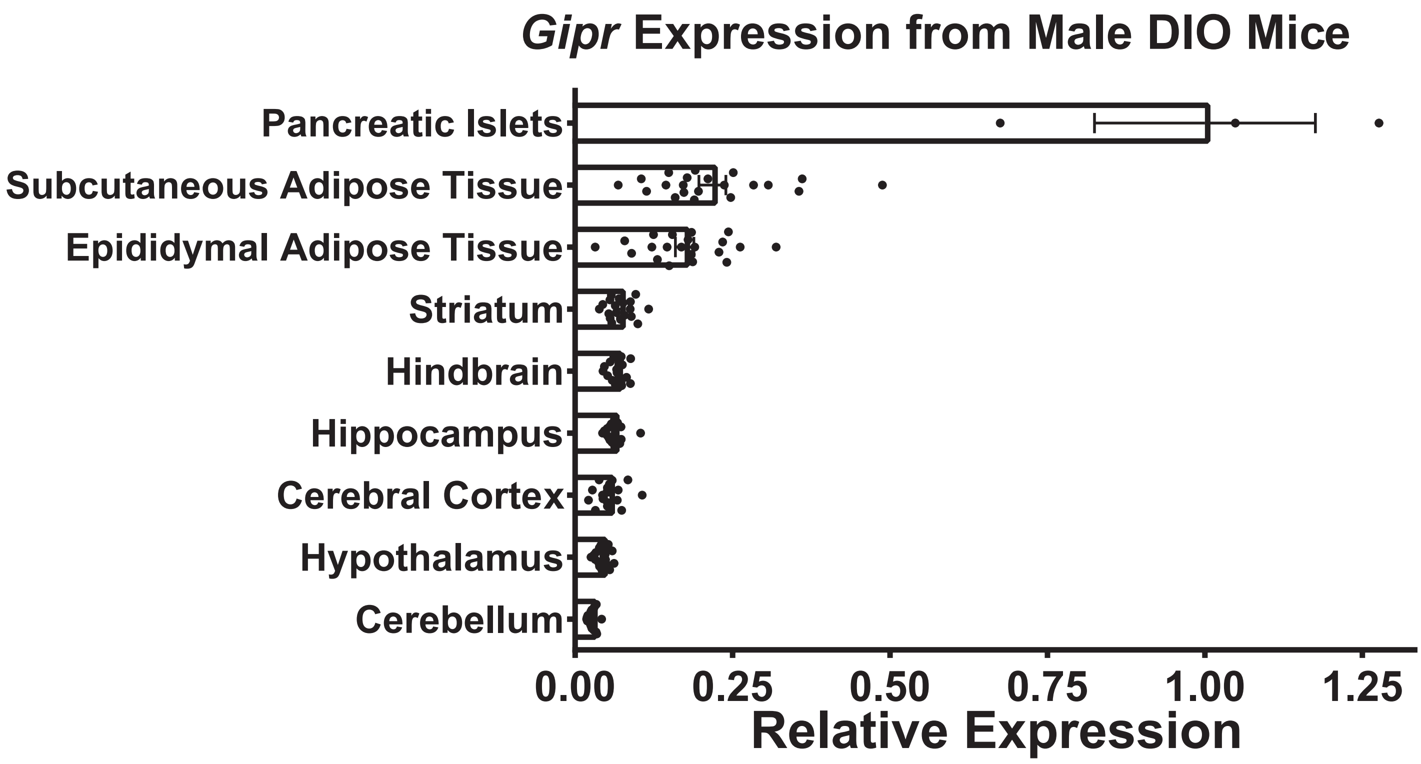

Supplemental Figure 5. Gipr Expression in pancreatic islets, adipose tissue, and brain sections from DIO mice.

Relative RNA expression of Gipr exons 9-10 relative to Eef2 in tissues from male DIO mice fed HFD for 12 weeks in isolated pancreatic islets (n = 3 pooled islets); white adipose tissue (WAT) and brain sections (n = 21 mice). Data are presented as mean values +/- SEM.

Supplemental Table and Legend

Supplemental Table 1. Mean noncompartmental pharmacokinetic parameter estimates following single IV or IP administration of LA-Agonist to CD-1 mice

Route	Dose (mg/kg)	N	$AUC_{0\text{-inf}}$ (hr*µg/mL)	CL or CL/F (mL/hr/kg)	$t_{1/2,z}$ (hr)	F (%)
IV	5	3	1510	3.32	66.7	--
IP	5	3	1580	3.18	71.3	~100

$AUC_{0\text{-inf}}$ = area under the plasma concentration-time curve from time 0 to infinity; CL = clearance after IV administration; CL/F = apparent CL after IP administration; F = bioavailability after IP administration; IP = intraperitoneal; IV = intravenous; N = number of animals; $t_{1/2,z}$ = half-life associated with the terminal phase.

Supplemental Data Legend

Supplemental Data 1 (Excel File). Metabolic profiling of plasma from DIO mice.

Heat map of statistically significant biochemicals profiled in this study. Red and green shaded cells indicate $p \leq 0.05$ (red indicates that the mean values are significantly higher for that comparison; green values significantly lower). Light red and light green shaded cells indicate $0.05 < p < 0.10$ (light red indicates that the mean values trend higher for that comparison; light green values trend lower). Standard statistical analyses were performed in ArrayStudio on log transformed data using Welch's two-sample t-test to test whether two unknown means are different from two independent populations with $p < 0.05$ indicating statistical significance. To correct for multiple comparisons, the q-value method for False Discovery
