# Supplementary material for: Chronic glucose-dependent insulinotropic polypeptide receptor (GIPR) agonism desensitizes adipocyte GIPR activity mimicking functional GIPR antagonism
Source: Nat Commun. 2020 Oct 5;11:4981. doi: 10.1038/s41467-020-18751-8 (PMC7536395; doi:10.1038/s41467-020-18751-8)
Supplement: Supplementary file 3 — Description of Additional Supplementary Files [file 41467_2020_18751_MOESM3_ESM.pdf]

Title: Supplemental Data 1. Metabolic profiling of plasma from DIO mice.

Description: Heat map of statistically significant biochemicals profiled in this study. Red and green shaded cells indicate $p \leq 0.05$ (red indicates that the mean values are significantly higher for that comparison; green values significantly lower). Light red and light green shaded cells indicate $0.05 < p <$ 0.05 indicating statistical significance. To correct for multiple comparisons, the q-value method for False Discovery
