# Supplementary material for: Chronic glucose-dependent insulinotropic polypeptide receptor (GIPR) agonism desensitizes adipocyte GIPR activity mimicking functional GIPR antagonism
Source: Nat Commun. 2020 Oct 5;11:4981. doi: 10.1038/s41467-020-18751-8 (PMC7536395; doi:10.1038/s41467-020-18751-8)
Supplement: Supplementary file 5 — Reporting Summary [file 41467_2020_18751_MOESM5_ESM.pdf]

nature research

Corresponding author(s): David Lloyd

Last updated by author(s): Sep 4, 2020

Reporting Summary

Nature Research wishes to improve the reproducibility of the work that we publish. This form provides structure for consistency and transparency in reporting. For further information on Nature Research policies, see Authors & Referees and the Editorial Policy Checklist .

Statistics

For all statistical analyses, confirm that the following items are present in the figure legend, table legend, main text, or Methods section.

n/a	Confirmed	
☐	☒	The exact sample size (n) for each experimental group/condition, given as a discrete number and unit of measurement
☐	☒	A statement on whether measurements were taken from distinct samples or whether the same sample was measured repeatedly
☐	☒	The statistical test(s) used AND whether they are one- or two-sided<br>*Only common tests should be described solely by name; describe more complex techniques in the Methods section.*
☐	☒	A description of all covariates tested
☐	☒	A description of any assumptions or corrections, such as tests of normality and adjustment for multiple comparisons
☐	☒	A full description of the statistical parameters including central tendency (e.g. means) or other basic estimates (e.g. regression coefficient) AND variation (e.g. standard deviation) or associated estimates of uncertainty (e.g. confidence intervals)
☐	☒	For null hypothesis testing, the test statistic (e.g. F, t, r) with confidence intervals, effect sizes, degrees of freedom and P value noted<br>*Give P values as exact values whenever suitable.*
☒	☐	For Bayesian analysis, information on the choice of priors and Markov chain Monte Carlo settings
☒	☐	For hierarchical and complex designs, identification of the appropriate level for tests and full reporting of outcomes
☒	☐	Estimates of effect sizes (e.g. Cohen's d, Pearson's r), indicating how they were calculated

Our web collection on statistics for biologists contains articles on many of the points above.

Software and code

Policy information about availability of computer code

Data collection	Microsoft Excel was used for data collection.
Data analysis	Data was analyzed using Graphpad Prism (version 7.02 or version 7.04).

For manuscripts utilizing custom algorithms or software that are central to the research but not yet described in published literature, software must be made available to editors/reviewers. We strongly encourage code deposition in a community repository (e.g. GitHub). See the Nature Research guidelines for submitting code & software for further information.

Data

Policy information about availability of data

All manuscripts must include a data availability statement. This statement should provide the following information, where applicable:

- Accession codes, unique identifiers, or web links for publicly available datasets
- A list of figures that have associated raw data
- A description of any restrictions on data availability

All data is provided in the Source Data excel file.

Field-specific reporting

Please select the one below that is the best fit for your research. If you are not sure, read the appropriate sections before making your selection.

☒ Life sciences ☐ Behavioural & social sciences ☐ Ecological, evolutionary & environmental sciences

For a reference copy of the document with all sections, see nature.com/documents/nr-reporting-summary-flat.pdf

Life sciences study design

All studies must disclose on these points even when the disclosure is negative.

Sample size	Sample size was determined using internal pilot studies or data previously published in the literature (Killion et al., Science Trans. Med., 2018)
Data exclusions	Outliers were excluded from a group if the measurement was ±2 SDs from the group mean (Figure 2J, K, and Figure 9H, K).
Replication	The outcomes of all in vitro work presented have been replicated at least twice and all attempts at replication were successful. For in vivo studies, outcomes have been replicated using alternative experimental models as presented here (i.e. GIP-stimulated fatty acid uptake in acute study, chronic study, and in both GiprBCell-/- and GiprAdipo-/-). Studies using the long-acting GIPR agonist (LA-Agonist) have not been replicated more than once because material availability is extremely limited due tocost and degree of difficulty for synthesis. Studies utilizing GiprAdipo-/- mice have been replicated at least twice and all attempts at replication were successful.
Randomization	Mice were randomized to treatment groups based on body weight so that all treatment groups had identical starting body weight.
Blinding	No studies were blinded because treatment was administered by the researcher collecting the data. For metabolomics analysis, all analysis was performed by Metabolon, who were blinded to the experimental treatments, and were not altered in any way by Amgen or the authors.

Reporting for specific materials, systems and methods

We require information from authors about some types of materials, experimental systems and methods used in many studies. Here, indicate whether each material, system or method listed is relevant to your study. If you are not sure if a list item applies to your research, read the appropriate section before selecting a response.

Materials & experimental systems

n/a	Involved in the study	
☐	☒	Antibodies
☐	☒	Eukaryotic cell lines
☒	☐	Palaeontology
☐	☒	Animals and other organisms
☒	☐	Human research participants
☒	☐	Clinical data

Methods

n/a	Involved in the study	
☒	☐	ChIP-seq
☒	☐	Flow cytometry
☒	☐	MRI-based neuroimaging

Antibodies

Antibodies used	(1) Mouse anti-mouse GIPR monoclonal antibody (muGIPR-Ab, Amgen) - in vitro studies concentration noted in figure legends, in vivo concentration 12.5 mg/mL (25 mg/kg)<br>(2) Mouse anti-human GIPR monoclonal antibody (MAB8210, R&D Systems) - 10 ug/mL<br>(3) Alexa-Fluor 647 conjugated anti-mouse secondary antibody (A32728, Thermo Fisher) - 4 μg/mL
Validation	(1) Killion et al., Science Translation Medicine, 2018.<br>(2) https://www.rndsystems.com/products/human-gipr-antibody-591853_mab8210<br>(3) https://www.thermofisher.com/antibody/product/Goat-anti-Mouse-IgG-H-L-Highly-Cross-Adsorbed-Secondary-Antibody-Polyclonal/A32728

Eukaryotic cell lines

Policy information about cell lines

Cell line source(s)	(1) HEK 293T and CHOK1 cells overexpressing GIPR, GLP1R, or glucagon receptor were generated at Amgen.<br>(2) Mouse Neuro-2a neuroblastoma cells were purchased from ATCC<br>(3) Rat INS1 832/13 insulinoma cells were purchased from EMD Millipore<br>(4) Mouse primary adipocytes were isolated from C57Bl6/J mice purchased from Jackson Laboratories<br>(5) Human primary adipocytes were purchased from Zen-Bio
Authentication	Overexpressing cell lines were authenticated by flow cytometry. All other cell lines were not authenticated.
Mycoplasma contamination	Overexpressing cell lines tested negative for mycoplasma contamination. All other cell lines were not tested.
Commonly misidentified lines<br>(See ICLAC register)	No commonly misidentified cell lines were used.

Animals and other organisms

Policy information about studies involving animals; ARRIVE guidelines recommended for reporting animal research

Laboratory animals	(1) C57Bl6/J male mice fed HFD starting 6 weeks old for different lengths of time as indicated<br>(2) Gipr floxed mice generated on C57Bl6/N background and crossed to mice expressing Cre recombinase driven by the rat insulin promoter (RIP-Cre) previously described were purchased from Jackson Laboratories or mice expressing Cre recombinase driven by the Adipoq gene (Amgen).<br>GiprBCell-/- male mice (C57Bl6/N background) were used as indicated and were fed HFD starting at 7 weeks old for different lengths of time as indicated.<br>GiprAdipo-/- both male and female mice (C57Bl6/N background) were used as indicated and were fed HFD starting at 7 weeks old for different lengths of time as indicated.
Wild animals	The study did not involve wild animals.
Field-collected samples	The study did not involve samples collected from the field.
Ethics oversight	All mice were maintained according to the regulations of the Amgen IACUC in Thousand Oaks, CA

Note that full information on the approval of the study protocol must also be provided in the manuscript.
